# The val^158^met COMT polymorphism's effect on atrophy in healthy aging and Parkinson's disease

**DOI:** 10.1016/j.neurobiolaging.2008.07.009

**Published:** 2010-06

**Authors:** J.B. Rowe, L. Hughes, C.H. Williams-Gray, S. Bishop, S. Fallon, R.A. Barker, A.M. Owen

**Affiliations:** a University of Cambridge, Department of Clinical Neurosciences, CB2 2QQ, UK; b Medical Research Council, Cognition and Brain Sciences Unit, 15 Chaucer Road, Cambridge CB2 7EF, UK; c Cambridge Centre for Brain Repair, CB2 OPY, UK

**Keywords:** COMT, Catechol-*o*-methyltransferase, VBM, Parkinson's disease, Healthy aging, MRI

## Abstract

We investigated whether the val^158^met functional polymorphism of catechol-*o*-methyltransferase influenced age-related changes in grey matter density and volume, both in healthy individuals (*n* = 80, ages 18–79) and those with Parkinson's disease (*n* = 50). Global grey matter volumes and voxelwise estimates of grey matter volume and density were determined from structural magnetic resonance images at 3 T. Male and female ValVal homozygotes (low prefrontal cortical dopamine) had more grey matter in early adulthood, but this difference disappeared with increasing age. The insula and ventral prefrontal cortex had higher grey matter volume in younger, but not older, ValVal homozygotes. Conversely, the dominant premotor cortex revealed genotypic differences in grey matter density in later life. There were no global or local interactions between Parkinson's disease and COMT val^158^met genotype on morphometry. Since the val^158^met polymorphism is associated with differences in cortical dopamine metabolism, our data suggest a role for dopamine in cortical development followed by differential vulnerability to cortical atrophy across the adult life span.

## 1 Introduction

Dopamine is one of the most important regulators of brain function. In adults it is closely associated with affective and executive cognitive functions, especially of the frontal lobes and basal ganglia, and it is central to the pathophysiology of Parkinson's disease and schizophrenia. Dopamine also influences cortical neuronal development (Krasnova et al., 2007; Sherren and Pappas, 2005) and apoptosis (Iwatsubo et al., 2007). This raises the possibility of structural as well as functional consequences of chronic differences in dopaminergic function.

Chronic differences in cortical dopamine arise in part from functional polymorphisms in the gene for membrane bound catechol-*o*-methyltransferase (COMT) the principal catabolic enzyme for cortical dopamine (Yavich et al., 2007). In contrast, COMT contributes little to striatal dopamine catabolism (Tunbridge et al., 2004). The substitution of valine (Val) by methionine (Met) at codon 158 causes a trimodal distribution of enzymatic activity: ValVal vs. ValMet vs. MetMet. Functionally, this is the most significant of the many COMT polymorphisms. There is evidence of structural effects of this val^158^met polymorphism in specific groups, e.g. Val-hemizygote men with velo-cardio-facial syndrome (van Amelsvoort et al., 2008) and adults with schizophrenia or a high risk of schizophrenia (McIntosh et al., 2007; Ohnishi et al., 2006).

In Parkinson's disease (PD) motor and cognitive symptoms are also associated with chronic dopamine abnormalities within the cortex (Nagano-Saito et al., 2004; Rakshi et al., 1999) even though striatal pathology develops ahead of cortical pathology (Braak et al., 2006). Even in early PD, the val^158^met COMT polymorphism influences executive functions that are mediated by frontal cortex (Williams-Gray et al., 2008). The question arises therefore whether there are common effects of the val^158^met polymorphism and PD, e.g. do the cortical dopamine hypermetabolism in early PD and the hyperdopaminergic state of MetMet homozygotes have similar influences on cortical structure, or do they interact?

The effects of PD and COMT must be interpreted in the light of age related changes in grey matter morphometry (Good et al., 2001). However, the effect of the val^158^met polymorphism on age-related atrophy has not yet been identified across the adult lifespan. We therefore studied total grey matter volume in adults with and without PD aged 18–79 years. We then analysed regional changes in grey matter volume and density. We hypothesised that the val^158^met polymorphism interacts with global and regional rates of atrophy. We secondly hypothesised that such interactions would be influenced by the chronic dopaminergic abnormalities in PD.

## 2 Methods

### 2.1 Subjects

Eighty-two healthy adults participated, with no current neurological or psychiatric history (bimodal age distribution: old: 51–78, mean 66.2, S.D. 7.3, MMSE 29.2; and young: 20–47, mean 25.6, S.D. 6.7). Fifty patients with Parkinson's disease were recruited from the Cambridge PD Research Clinic (UK Parkinson's Disease Society Brain Bank clinical diagnostic criteria), 50–80 years (mean 64.9, S.D. 9); with no current depression (Beck Depression Inventory II score <13) or dementia (clinical diagnostic criteria and recent MMSE ≥27). See supplementary Table S1 for patient subgroup characteristics.

### 2.2 Genotype

DNA was extracted from peripheral blood samples using standard phenol/chloroform methods (Williams-Gray et al., 2007). The val^158^met genotype was determined by allelic discrimination TaqMan assays. This generates an allele-specific fluorescent reporter signal using the 5′ exonuclease activity of *Taq* polymerase. This signal was measured after PCRs using the HT7900 detection system (Applied Biosystems, Foster City, CA).

### 2.3 MRI data

MRI used a high resolution MPRAGE sequence (TR 2250 ms, TE 2.99 ms, FA 9 degrees, IT 900 ms, 256 × 256 × 192 isotropic 1 mm voxels) with a Siemens Tim Trio 3 Tesla scanner. Pre-processing used SPM5 software (fil.ion.ucl.ac.uk/spm) and its VBM toolbox (dbm.neuro.uni-jena.de/vbm) with Matlab 7 (Mathworks, USA). Data underwent an iterative unified segmentation normalisation procedure (Ashburner and Friston, 2005) into three tissue segments (GM, WM, CSF) with bias correction and warping to the Montreal Neurological Institute T1 template. The Hidden Markov Random Fields model was applied to constrain voxelwise segmentation based on nearest neighbours (Cuadra et al., 2005). The normalised modulated and unmodulated images of grey matter (GM) were smoothed by an isotropic Gaussian kernel (FWHM 16 mm).

Global GM volume was analysed in SPSS 11.0 (Chicago, Ill). We used a stepwise linear regression model including age, sex, val^158^met genotype, disease (PD vs. control), the 2-way interactions (‘age × genotype’, ‘age × sex’, ‘genotype × sex’, ‘disease group × age’, ‘disease group × genotype’) 3-way interactions and a constant term.

Voxel based morphometry (Ashburner and Friston, 2000) used general linear models to characterise the effects of COMT (ValVal, ValMet or MetMet), age and PD on differences in regional grey matter density (unmodulated images) and volume (modulated images). The models included regressors that separately specified each COMT subgroup (ValVal, ValMet, MetMet) for patients, older controls (>50) and younger controls (<50)(see supplementary Fig. S1) and total GM volume as a covariate. In addition, linear and quadratic functions of the UPDRS were included for each subgroup of patients.

Because of the predominant frontal distribution of cortical dopamine and the evidence of COMT modulation of frontal cortical functions, we restricted our study to a frontal lobe region of interest (supplementary Fig. S2) including all the frontal lobe regions and striatum as defined by Anatomical Automatic Labelling in MNI space (Tzourio-Mazoyer et al., 2002). We corrected for local smoothness and multiple comparisons (family wise error rate FWE *p* < 0.05). Additional results of interest are presented at the secondary threshold *p* < 0.001 unc.

## 3 Results

### 3.1 Global GM volume

**Fig. 1:**
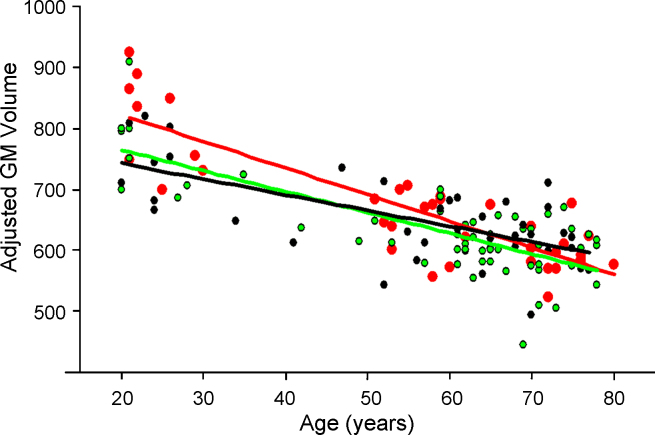
Global GM volume changes with age in ValVal (red), ValMet (green) and MetMet (black) groups for healthy subjects and patients with Parkinson's disease (adjusted for gender differences). Lines indicate least squares linear regression trends.

Fig. 1 shows the adjusted GM volume changes with age for the three genotypic groups of participants: ValVal, ValMet and MetMet. Stepwise linear regression indicated three significant variables: age (*t*(126) = −14.0, *p* < 0.001, rate = 0.4% per year), sex (*t*(126) = −6.60, *p* < 0.001, 6.7% difference) and the interaction between age and genotype (*t*(126) = 2.28, *p* < 0.05, global atrophy rate difference between ValVal and MetMet 0.16% per year) accounting for 67% of GM volume variance (*r* = 0.823, *F* 3,126 = 88, *p* < 0.001). Excluded factors had *t*-statistics in the range −0.6 < *t* < 0.6 (*p* > 0.5) except ‘disease × age’ (*t* = −1.3, *p* = 0.20). The tolerances (>0.99) and variance inflation factors (<1.01) were good.

A post hoc unpaired *t*-test of sex adjusted GM data from young subjects (age < 50) confirmed that ValVal subjects had more GM volume than MetMet subjects (*t*(17) = 2.32, 2-tailed *p* < 0.05). Because of collinearity between disease (PD) and age, a subsidiary analysis was run using data from the older subjects only (age > 50, *n* = 100). This supported a reduced model that included just age and sex as significant factors. Age was highly significant (*t*(98) = −3.74, *p* < 0.001, effect 0.36% per year) as was sex (*t*(98) = −5.73,*p* < 0.001, 8.2% difference). The effects of ‘age × genotype’ (*t*(98) = −0.92) disease (*t*(98) = −0.05) and genotype (*t*(98) = 0.2) were not significant.

### 3.2 Regionally specific changes in GM density

There were extensive differences between older and younger healthy subjects collapsed across genotype (contrast [zeros(1,9) −1 −1 −1 1 1 1]). Younger subjects had higher density in medial and lateral prefrontal cortex, dorsally and ventrally, and the caudate nuclei (supplementary Fig. S3A).

**Fig. 2:**
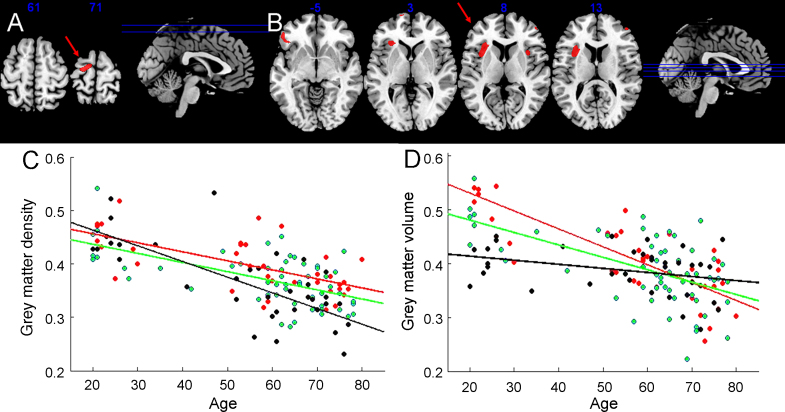
SPM{*t*} maps of voxelwise morphometric interactions in healthy subjects between age and COMT genotype. (A) GM density differences increasing with age (FWE *p* < 0.05) and (B) GM volume differences reducing with age (*p* < 0.001 unc.). Peaks of interest are indicated by the red arrows. (C) GM densities in premotor cortex (MNI *x*,*y*,*z* = −12 −8 71) are plotted against age for ValVal (red), ValMet (green) and MetMet (black) groups. (D) GM volume in the left frontal operculum/insula (MNI *x*,*y*,*z* = −33, 28, 1) are plotted against age for ValVal (red), ValMet (green) and MetMet (black) groups.

Across the whole group, there were no significant regional differences between ValVal vs. MetMet groups (SPM{*t*}contrast ± [1 0 0 0 0 0 −1 0 0 1 0 −1 1 0 −1] or SPM{F} contrast [1 0 0 0 0 0 −1 0 0 1 0 −1 1 0 −1; 0 0 0 1 0 0 −1 0 0 0 1 −1 1 0 −1]). An interaction between genotype and aging was identified in the left premotor cortex (contrast [1 0 0 0 0 0 −1 0 0 1 0 −1 −2 0 2], peak −14,−10,70, *t* = 3.56, *p* < 0.001, Fig. 2A). This interaction was driven by differences between older ValVal and MetMet subjects (>50 years) (contrast [1 0 0 0 0 0 −1 0 0 1 0 −1 0 0 0] peak *x*,*y*,*z* = −12 −8 71, *t* = 4.42, FWE *p* < 0.05, Fig. 2C). This suggests that with aging, subjects with MetMet genotype had lost grey matter density from the dominant hemisphere premotor region faster than was proportionate to global atrophy (Fig. 2C). Frontal lobe GM density did not differ between patients and older controls (contrast [1 0 0 1 0 0 1 0 0 −1 −1 −1 0 0 0]) and there were no differences related to linear or quadratic functions of disease severity (UPDRS) nor PD × genotype interactions (±[1 0 0 0 0 0 −1 0 0 −1 0 1 0 0 0]).

### 3.3 Regionally specific changes in GM volume

There were extensive differences between old and young healthy subjects, collapsing across the genotypes ([0 0 0 0 0 0 0 0 0 −1 −1 −1 1 1 1], supplementary Fig. S3C). Older subjects had regional loss of GM volume in the supplementary motor area (1, −13, 73, *t* = 5.03, FWE *p* < 0.05), pre-supplementary motor area (1, 20, 64, *t* = 5.01, FWE *p* < 0.05) and medial prefrontal cortex (1, 42, 32, *t* = 7.45, FWE *p* < 0.05), bilateral polar cortex (±30, 56, 26, *t* = 4.87, FWE *p* < 0.05), ventrolateral prefrontal cortex (53,31,−13, *t* = 5.62, FWE *p* < 0.05; −54, 19, 28, *t* = 5.11, FWE *p* < 0.05) and caudate (−3, 6, 7, *t* = 6.63; 6,9,9,*t* = 5.22, FWE *p* < 0.05).

There were weak regional effects of genotype amongst healthy subjects ([0 0 0 0 0 0 0 0 0 1 0 −1 1 0 −1], see supplementary Table S2) and interactions between genotype and age. Specifically, there were bilateral volume differences (ValVal > MetMet) in the anterior insula and ventral frontal cortex in young subjects (young subjects <50 years: ValVal > MetMet difference in the left frontal operculum/anterior insula, [0 0 0 0 0 0 0 0 0 0 0 0 1 0 −1], peak −33, 28, 1, *t* = 4.97, FWE *p* < 0.05) with an interaction between age and genotype ([0 0 0 0 0 0 0 0 0 −1 0 1 1 0 −1] peak 32, 27,1, *t* = 4.18, *p* < 0.001 unc., see Fig. 2B). In the older subpopulations (>50 years) there were volume differences by genotype (ValVal > MetMet) in left premotor cortex (−12, −8, 72, *t* = 4.06, *p* < 0.001 unc.).

PD was associated with GM volume loss in the medial frontal cortex (peak −2, 36, 50, *t* = 4.34, FWE *p* < 0.05, supplementary Fig. S3D) extending caudally (at *p* < 0.001 unc.) to pre-supplementary motor area (5,6,44, *t* = 3.95) with additional peaks in precuneus (−3, −66, 46, *t* = 3.87) and visual cortex (−2, −91, 1, *t* = 3.71). There were no significant differences related to the linear or quadratic functions of disease severity (UPDRS) nor PD × genotype interaction (±[1 0 0 0 0 0 −1 0 0 −1 0 1 0 0 0]).

## 4 Discussion

Global GM volume was greater in young ValVal homozygotes consistent with previous reports of Val vs. Met hemizygotes, and despite previous negative reports for adult homozygotes (Ohnishi et al., 2006; Zinkstok et al., 2006). However, our data do not provide evidence of continuing differences in ValVal homozygotes. Instead, the genotype effect on global GM diminished with increasing age, suggesting that the global developmental differences arising from the COMT val^158^met polymorphism are lost during adulthood.

These cortical structural differences may contribute to the complex changes in executive function with normal aging. Across the adult age span (35–85 years) ValVal homozygotes have a faster decline in executive function over short intervals (de Frias et al., 2005) but by old age (>65 years) the val^158^met genotype has less influence (de Frias et al., 2005; Starr et al., 2007). Our data suggest that the loss of this effect of COMT genotype on executive function with age may be due in part to the differential loss of GM although the mechanism for this vulnerability to aging is currently unknown. It is possible that the ValVal associated accelerated atrophy with healthy aging is mechanistically associated with the ValVal associated atrophy in schizophrenia (McIntosh et al., 2007; Ohnishi et al., 2006) but further work is required to establish such a link. However, in contrast to schizophrenia, idiopathic PD did not interact with the effects of val^158^met COMT genotype.

We also identified regional differences in the frontal lobes, in terms of GM density and volume related to the val^158^met genotype and interactions between age and genotype. These structural differences partially correlate with regional differences in function. For example, fMRI studies have shown that the val^158^met polymorphism affects the frontal activations during tasks of executive function in PD (Williams-Gray et al., 2007) and health (Egan et al., 2001; Meyer-Lindenberg et al., 2005). Typically there is greater activation in lateral or medial prefrontal cortex at equivalent levels of performance for healthy ValVal adults. Of these regions, the ventral lateral prefrontal cortex is associated with structural genotypic differences in grey matter in the current study.

Parietal activity also differs between ValVal and MetMet adults during some executive tasks (Williams-Gray et al., 2007). However, the current structural data (supplementary Table S2) and previous functional data of regional blood flow (Meyer-Lindenberg et al., 2005) suggest that the parietal difference is secondary to the frontal effects of genotype, with fronto-parietal connections, rather than a direct influence on parietal cortical activity.

Clearly, there are limitations to the study. Our sample size (*n* = 130) is generally sufficient for VBM but we have several subgroups including medicated patients. Although our PD population was larger than most previous VBM studies of parkinsonian syndromes, and we reproduce VBM effects of PD, we did not find an interaction between disease and COMT genotype. This might be a false negative result, due to small effect size. However, we cannot rule out two further possibilities: a compensatory frontal cortical *hyper-*dopaminergic state in early PD (Rakshi et al., 1999) or that dopaminergic medication obviates structural changes arising from PD itself. Therefore, independent replication of our findings would be helpful, especially with unmedicated patients. Finally, it should be noted that we have reported the complementary information about human cortical change, including atrophy, given by VBM of both modulated and unmodulated brain images. However, VBM does not indicate whether these differences arise from changes to the neuron soma, the neuropil or even glia.

In summary, our data indicate age-related changes in global and regional grey matter with the val^158^met polymorphism of COMT. These may be of functional significance in executive and affective aspects of cognitive aging. We suggest that functional effects of the val^158^met polymorphism may be partly due to differences in the efficacy of current dopamine synaptic transmission, but also differences in local cortical structure.

## Conflict of interest

The authors have no conflict of interest.
