# Supplementary material for: The val158met COMT polymorphism's effect on atrophy in healthy aging and Parkinson's disease
Source: Neurobiol Aging. 2010 Jun;31(6):1064–8. doi: 10.1016/j.neurobiolaging.2008.07.009 (PMC3898476; doi:10.1016/j.neurobiolaging.2008.07.009)
Supplement: Supplementary file 1 [file mmc1.pdf]

Supplementary material

Supplementary table S1: Demographic and clinical characteristics of the study populations by val^{158}met COMT genotype. Values indicate means (standard deviation in parenthesis) except for the Male to Female ratios M:F.

Genotype	ValVal	ValMet	MetMet
**PD patient groups**			
M:F	11:5	10:8	9:6
Age	64.3 (9.8)	65.1 (7.7)	65.5 (9.6)
MMSE	28.9 (1.2)	28.3 (1.6)	28.7 (0.8)
BDI	6.8 (4.3)	8.6 (4)	6/.7 (4.3)
NART estimated IQ	113 (7)	108 (11)	114 (6)
Years from diagnosis	3.9 (2)	7.8 (3.5)	3.4 (1.5)
UPDRS ‘on’	22 (10)	20 (8)	26 (9)
l-dopa, mg/d equivalent	620 (480)	1330 (500)	620 (430)
**Control group (all)**			
M:F	13:9	18:18	13:9
Age (SD)	49 (22)	55 (20)	47 (19)
**Control group (>50 years)**			
M:F	8:5	14:12	8:4
Age (SD)	67 (8)	66 (7)	63 (7)

Patients were examined on their usual medication using the Unified Parkinson’s Disease Rating Scale (UPDRS, motor subscale III) and the Folstein Mini-Mental-State-Examination (MMSE). BDI = Beck Depression Inventory –II completed between assessments. There were more men than women in the patient groups (Chi-sq=9, df 2, P<0.01: M>F) but this inequality was not significantly different from the older control group (Chi-sq =5.4, df 5, p>0.1). 3/132 subjects were excluded due to significant structural abnormalities on MRI.

Supplementary Table S2. Peaks of regional differences in grey matter volume between ValVal and MetMet healthy subjects, adjusted for global differences in GM volume (SPM{t} contrast [zeros(1,9) 1 0 -1 1 0 -1], at threshold p<0.001 unc. See also figure 2B). Coordinates are with reference to standard anatomic space using the Montreal Neurological Institute template. t = t-statistic; l = left hemisphere; r = right hemisphere.

Region		x	y	x	t
Anterior insula	l	-34	11	11	4.06
	l	-31	27	3	3.64
	r	36	12	8	3.49
Lateral prefrontal cortex	l	-50	38	-7	3.78
	l	-46	30	-8	3.99
	r	42	33	31	3.43
Polar prefrontal cortex	l	-15	69	2	3.40
	r	48	50	10	3.55

Supplementary Figure S1. The design matrix used for the principal voxel based morphometric analyses of GM volume and density. Patients are characterised by genotype (ValVal column 1, ValMet column 4 and MetMet column 7) and for each genotype the normalised UPDRS (columns 2, 5, 8) and quadratic function of UPDRS (columns 3, 6, 9). The three genotype subgroups of healthy older subjects are represented by columns 10-12 (ValVal column 10, ValMet column 11 and MetMet column 12), and the three genotype populations of healthy younger subjects are represented by columns 13-15 (ValVal column 13, ValMet column 14 and MetMet column 15). The total GM volume is included as a covariate (column 16) such that VBM results reflect local not global changes in grey matter.

Supplementary Figure S2. The reduced search volume defined for the frontal lobe and striatum, overlaid on a representative brain in standard anatomic space using the Montreal Neurological Institute template (z- and y-value of slices are shown in blue). Top row: coronal slices. Bottom row, axial slices. Right, 3-D rendering viewed from front left hand side.

Supplementary Figure S3. SPM{t} maps of voxelwise morphometric changes in GM density (A,B) and volume (C, D) related to age (A,C), and Parkinson's disease (B,D). Voxels shown in red are above the t-threshold for which $p<0.05$ in the frontal lobe search volume (family wise error corrected for multiple comparisons). The SPM{t} contrast images are overlaid on a representative brain in standard anatomic space using the Montreal Neurological Institute template (z-value of slices is shown in blue).
